# Correction: Günlü, O. Function Computation under Privacy, Secrecy, Distortion, and Communication Constraints. *Entropy* 2022, *24*, 110

**DOI:** 10.3390/e24070861

**Published:** 2022-06-23

**Authors:** Onur Günlü

**Affiliations:** Chair of Communications Engineering and Security, University of Siegen, 57076 Siegen, Germany; onur.guenlue@uni-siegen.de

Special case results given in Lemmas 1–4 and evaluated in Section 4.4 as an example are unfortunately not necessarily the exact rate regions for all channel models, although they are valid inner bounds. In more detail, the inner bounds given in Theorems 1 and 2 can be simplified for functions that are partially invertible with respect to $\tilde{X_{j}}$ by choosing the auxiliary random variable $U_{j}=\tilde{X_{j}}$ since $U_{j}^{n}$ represents a distorted version of ${\tilde{X_{j\;}}}^{n}$. Similarly, for invertible functions one can simplify the inner bounds by choosing $U_{1}=\tilde{X_{1}}$ and $U_{2}=\tilde{X_{2}}$. However, for neither special case it is necessarily optimal to choose the other auxiliary random variables V_1_ and V_2_ as constants, since they represent a step that aims to increase the equivocation at the eavesdropper. Therefore, we replace the wordings for all special cases considered in [1] by stating that the rate regions given in Lemmas 1–4 and evaluated in Section 4.4 are valid and computable inner bounds for the corresponding special cases for all channel models. These changes can be implemented in [1] by replacing:

1. “exact lossless” in the Abstract and Sections 1.2, 1.3 and 4.1 with “simplified lossless”.
2. “exact rate region” in Sections 1.2, 1.3, 3.2, 4, 4.1, 4.2 and 6 with “simplified rate region”.
3. “The … rate region … when … is the set of …” in Lemmas 1–4 with “The … rate region … when … includes the set of …”.
4. “the lossless rate regions” in Section 4.3 with “the further simplified lossless rate regions”.
5. “the exact lossless rate region” in Section 4.3.1 with “the further simplified lossless rate region”.
6. “the lossless rate region” in Sections 4.3.2 and 4.4 with “an inner bound for the lossless rate region”.
7. “one exact region” in the Abstract with “one achievable region”.
8. “evaluate the exact rate region” in Section 1.2 with “evaluate a simplified rate region”.
9. “the rate region for” in Section 1.3 with “a rate region for”.
10. “the exact lossless” in Section 4.3.2 with “a simplified lossless”.

Furthermore, below Remark 1, delete the sentence “i.e., rate regions for which inner and outer bounds match”. Since [1] is an extension of [32,33], the same changes are relevant also for [32] (Corollaries 1 and 2) and [33] (Corollary 1).

The author states that the scientific conclusions are unaffected. This correction was approved by the Academic Editor. The original publication has also been updated.
